# Thermodynamic Stability Is a Strong Predictor for the Delivery of DARPins to the Cytosol via Anthrax Toxin

**DOI:** 10.3390/pharmaceutics13081285

**Published:** 2021-08-18

**Authors:** Lukas Becker, Jasleen Singh Badwal, Fabian Brandl, Wouter P. R. Verdurmen, Andreas Plückthun

**Affiliations:** 1 Department of Biochemistry, University of Zurich, Winterthurerstr. 190, 8057 Zurich, Switzerland; l.becker@bioc.uzh.ch (L.B.); j.singhbadwal@bioc.uzh.ch (J.S.B.); brandl.fabian@gmail.com (F.B.); 2 Department of Biochemistry, Radboud Institute for Molecular Life Sciences (RIMLS), Radboud University Medical Center, Geert Grooteplein 28, 6525 GA Nijmegen, The Netherlands; wouter.verdurmen@radboudumc.nl

**Keywords:** anthrax toxin, cytosolic protein delivery, DARPin, circular dichroism, protein stability

## Abstract

Anthrax toxin has evolved to translocate its toxic cargo proteins to the cytosol of cells carrying its cognate receptor. Cargo molecules need to unfold to penetrate the narrow pore formed by its membrane-spanning subunit, protective antigen (PA). Various alternative cargo molecules have previously been tested, with some showing only limited translocation efficiency, and it may be assumed that these were too stable to be unfolded before passing through the anthrax pore. In this study, we systematically and quantitatively analyzed the correlation between the translocation of various designed ankyrin repeat proteins (DARPins) and their different sizes and thermodynamic stabilities. To measure cytosolic uptake, we used biotinylation of the cargo by cytosolic BirA, and we measured cargo equilibrium stability via denaturant-induced unfolding, monitored by circular dichroism (CD). Most of the tested DARPin cargoes, including target-binding ones, were translocated to the cytosol. Those DARPins, which remained trapped in the endosome, were confirmed by CD to show a high equilibrium stability. We could pinpoint a stability threshold up to which cargo DARPins still get translocated to the cytosol. These experiments have outlined the requirements for translocatable binding proteins, relevant stability measurements to assess translocatable candidates, and guidelines to further engineer this property if needed.

## 1. Introduction

Anthrax toxin is an AB-type bacterial holotoxin of *Bacillus anthracis*. It comprises two protein components with distinct functions: a catalytically active and toxic A component that relies on the cell-binding and pore-forming B component, protective antigen (PA). There are two toxic A components: lethal factor (LF) and edema factor (EF). Upon cellular binding of PA, furin or furin-like proteases induce oligomerization of PA by cleaving off the 20 kDa domain 1. Three or four LF or EF moieties are able to bind to the oligomerized PAs, the prepore, consisting of seven or eight PA_63_ subunits [1]. This LF- and EF-bound prepore complex is taken up by the cells via clathrin-mediated endocytosis [2]. Due to the pH shift within the endosome, the PA prepore restructures and inserts a β-barrel-like pore into the membrane, which helps to unfold and translocate the bound LF and EF cargo through the pore [3].

The modular structure of anthrax toxin enabled us and others to generate engineered PA variants with altered cell specificity and the capability to translocate alternative LF cargo molecules [4,5,6,7,8,9]. Our group and others [6,10,11,12,13] showed that cytosolic translocation by pore-forming toxins depends on the ability of the molecule to fit through this channel. Therefore, multiple factors block translocation, such as unfolding influenced by protein cargo stability [11]; the presence of disulfide bridges [14]; strong negative charges that cannot be temporarily protonated, such as cysteic acid (pK_a_ = −1.9) [15]; and general bulkiness, as exemplified for nonprotein cargoes by cyclic peptides or a bulky small-molecule drug such as docetaxel [16]. The inability of anthrax toxin to deliver those protein cargo molecules thus lies in the prerequisite of cargo unfolding for the translocation, as passage through the PA pore is catalyzed by the narrow 6 Å clamp consisting of Phe residues, termed a phi (Φ)-clamp, via a charge-state-dependent Brownian ratchet [17,18,19].

Using the biotin ligase assay [20] to quantify cytosolically delivered cargo molecules, we investigated quantitatively to what extent the thermodynamic stability of the cargo is a predictor for successful delivery via PA pores. Specifically, our aim was to evaluate how stable a cargo molecule can be yet still be efficiently translocated. To systematically evaluate this relationship, we used designed ankyrin repeat proteins (DARPins), for which there is a great variety of potential cargo proteins available with different sizes and stabilities to define a potential stability translocation threshold. We show here that DARPin cargo proteins up to a ΔG_0_ value of around 10 kcal mol^−1^, representing the change in Gibbs free energy between the folded and unfolded states, can be translocated very efficiently to the cytosol, with more stable cargoes progressively demonstrating a poorer cytosolic translocation. Furthermore, we show that DARPins can be rationally destabilized to reach this threshold and are then efficiently translocated, without losing their folded structure or binding affinity.

## 2. Materials and Methods

### 2.1. Cell Lines

Flp-In 293 cells, stably overexpressing EpCAM and BirA (Flp-In 293-EpCAM-BirA [6]), were cultured using DMEM. The medium was supplemented with 10% fetal calf serum, 100 IU/mL penicillin, and 100 µg/mL streptomycin.

### 2.2. Cloning

Cloning of most constructs used in this study has been described before [6,9]. DARPin cargoes were cloned into the SpeI/AgeI-restricted pQIq-LF_N_-cargo-avi-HA backbone for protein delivery. For CD spectroscopy, DARPins were cloned via SpeI/AgeI in a pQIq expression vector without LF_N_, avi, or HA-tag.

### 2.3. Protein Expression

Expression of His_6_-MBP-PA_wt_-sANTXR-Ac2 and His_6_-MBP-LF_N_-cargo constructs has been described before [6,9]. For the expression of DARPins for CD spectroscopy, the *E. coli* strain BL21 was transformed with described plasmids. A single clone was picked the next day and used for an overnight culture in LB medium, supplemented with 100 µg/mL ampicillin at 37 °C. A 500 mL volume of LB medium, supplemented with 100 µg/mL ampicillin, was inoculated with 10 mL overnight culture and grown at 37 °C until an OD_600_ of 0.8 was reached. Protein expression was induced with 500 µM IPTG and incubated for 4 h at 30 °C. Cultures were centrifuged for 10 min at 5000 *g* at 4 °C, the pellet washed with PBS, pH 7.4, snap-frozen, and stored at −20 °C until purification.

### 2.4. Protein Purification

Purifications of His_6_-MBP-PA_wt_-sANTXR-Ac2 and His_6_-MBP-LF_N_-cargo constructs have been described before [9]. All unfused DARPins were purified in a similar manner at 4 °C. Tris-HCl buffers were adjusted to pH 8.0. Bacterial cell pellets were thawed and resuspended in lysis buffer (50 mM Tris-HCl, 1 mM EDTA, 500 mM NaCl, 10% glycerol), supplemented with 0.4 mM 4-(2-aminoethyl) benzenesulfonyl fluoride (AEBSF). 100 µg/mL DNase I and 1 g/L lysozyme were added. Cells were lysed by sonication and centrifuged for 45 min at 20,000 *g*. DARPins were purified by their His-tag via immobilized metal ion affinity chromatography (IMAC). HisPur^TM^ Ni-NTA Resin (Thermo Scientific) was packed in 2 mL benchtop columns (PD10), and columns were equilibrated in lysis buffer. Lysate was applied to the column, washed with 10 column volumes (CV) high-salt buffer (25 mM Tris-HCl, 500 mM NaCl, 20 mM imidazole), 10 CV low-salt buffer (25 mM Tris-HCl, 125 mM NaCl, 20 mM imidazole), and eluted with 2.5 CV elution buffer (25 mM Tris-HCl, 125 mM NaCl, 300 mM imidazole). DARPins were dialyzed overnight against PBS, snap-frozen in liquid N_2_, and stored short term at −20 °C.

### 2.5. Biotin Ligase Uptake Assay

To quantitate the total cellular uptake, as well as the cytosolic localization of cargo proteins, the biotin ligase assay was performed as described previously [20]. Cytosolic uptake was determined by normalizing signal intensities of cytosolic uptake to the signal intensity of the unspecific interaction of streptavidin with HSP70, which correlates well with the actin signal [6]. To cross-compare the quantification from different delivery assays, we normalized the quantified signals to the control (Cells only, 0%) and defined the signal for NI_1_C, the smallest and best-delivered DARPin, as 100%.

### 2.6. CD Spectroscopy and Equilibrium Unfolding

Proteins were diluted in PBS with increasing concentrations of GdnHCl to a final protein concentration of 10 µM. For CD spectroscopy analysis of DARPins at various pH values, DARPins were diluted in 50 mM of the respective buffer (MES at pH 6.5 and pH 6.0, sodium acetate at pH 5.5, pH 5.0, pH 4.5 and pH 4.0 and citric acid at pH 3.5). All buffers were supplemented with 150 mM NaCl. For CD spectroscopy analysis of DARPins at pH 6, a buffer exchange was performed, prior to GdnHCl dilution, via PD10 columns (GE Healthcare) to 50 mM MES (pH 6.0) and 150 mM NaCl.

Protein-GdnHCl solutions and protein solutions in different buffers were incubated overnight at room temperature for the systems to equilibrate. The CD signal at 222 nm was recorded on a Jasco J-715 instrument (Jasco, Japan) using a cylindrical quartz cell of 1 mm pathlength, 1 nm bandwidth, 4 s response time and 3 accumulations. Measurements were baseline-corrected to the respective buffer and converted to mean residue ellipticity (MRE). A nonlinear, least-squares fit was used (Equation (1)) to fit the unfolding curves and determine ΔG_0_ and m.

$$y_{obs}=\frac{\left(y_{f}+m_{f}\left[D\right]\right)+\left(y_{u}+m_{u}\left[D\right]\right)e^{\frac{-\Delta G_{H_{2}O}-m\left[D\right]}{RT}})}{1+e^{\frac{-\Delta G_{H_{2}O}-m\left[D\right]}{RT}}}$$

Here $y_{f}$, $m_{f}$, $y_{u}$, and $m_{u}$ describe the slope and intercept of the pre- and post-transition baselines. The transition region is characterized by $\Delta G_{H_{2}O}$ and m. The slopes of the baselines, $m_{f}$ and $m_{u}$, were restrained to be zero. The denaturation midpoint (*D_m_*) was calculated with Equation (2):

$$D_{m}=\frac{\Delta G_{H_{2}O}}{m}$$

Values for $\Delta G_{H_{2}O}$ and m were obtained from the fits using Equation (1). If fitting Equation (1) was not possible, i.e., when the protein did not behave as a two-state system, *D_m_* was estimated simply by a nonlinear (if required biphasic) fit (GraphPad Prism 8.0; X is concentration) to experimental data, to guide the eye. Values for *D_m_* estimated by this latter fit are clearly labeled throughout this manuscript.

### 2.7. Surface Plasmon Resonance (SPR)

Binding kinetics of off7 and off7 dest1 were determined as previously described [21]. A 1:1 Langmuir binding model was fit to the data measured on a ProteOn^TM^ XPR36 instrument (BioRad Laboratories, Hercules, CA, USA) with a ProteOn^TM^ NLC Sensor Chip (BioRad) using ProteOn^TM^ Manager Software (Version 3.1.0.6, BioRad).

## 3. Results

### 3.1. Cytosolic Translocation of LF_N_-DARPin Cargoes via PA_wt_-sANTXR-Ac2

Previously, we showed the delivery of consensus DARPins to the cytosol of Flp-In 293-EpCAM-BirA cells stably overexpressing the epithelial cell adhesion molecule (EpCAM) [6,9]. We developed PA_wt_-sANTXR-Ac2, a rationally designed PA variant retargeted to EpCAM [9], which does not prematurely form pores in the plasma membrane and can thus transport significantly more cargo to the cytosol than previous targeted versions. The translocation capability of PA_wt_-sANTXR-Ac2 (and of previous targeted PA variants) was limited to those consensus DARPins with low enough thermodynamic stability, but no quantitative assessment was carried out. Consensus DARPins with two or three internal repeats could be efficiently translocated only upon introducing destabilizing mutations in the DARPin framework, as consensus DARPins were originally engineered for very high stability [6,22]. However, no threshold value of equilibrium stability had been determined. In these consensus molecules, surface residues additionally contribute to the stability through charge interactions. In contrast, target-selected binders have a more varied surface and usually still have high stability, but not as high as the consensus molecules [23], and thus it was important to establish the transport capability of target-binding DARPins as well.

Here, we investigated the relation between DARPin cargo equilibrium stability and the translocation ability of PA_wt_-sANTXR-Ac2 with different cargo DARPins. Cargo DARPins were fused to the C-terminus of the PA-binding domain of lethal factor 1–254 (LF_N_), and they contain the biotin-acceptor avi-tag and an HA-tag at their C-terminus. LF_N_-cargoes consisted of consensus DARPins, LoopDARPins, and target-selected DARPins, and they varied in their number of internal repeats and therefore in size and thermodynamic stability.

We utilized the biotin ligase assay to quantify cytosolic delivery, a Western-blot-based method we previously published [20]. The biotin ligase assay relies on the stable overexpression of a cytosolically localized biotin ligase derived from *E. coli* (BirA) and the presence of an avi-tag on the cargo of interest, which gets biotinylated only when the cargo is present in the cytosol, as it requires direct contact with the resident biotin ligase. To perform this assay, we incubated Flp-In 293-EpCAM-BirA cells with PA_wt_-sANTXR-Ac2 and the respective cargo DARPins, fused to LF_N_, for 4 h in the presence of the proteasome inhibitor MG-132. Cells incubated without PA_wt_-sANTXR-Ac2 or LF_N_-cargo fusions were collected as controls. We used an LF_N_-eGFP fusion as a negative control for delivery due to endosomal entrapment of this molecule, as it cannot be unfolded and pass through the pore, as reported previously [9].

**Figure 1:**
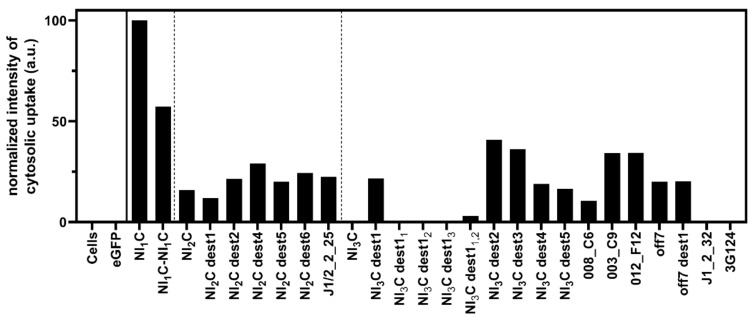
Quantification of Western blots shown in Supplementary Figure S1, measuring the delivery of different LF_N_-cargo constructs with PA_wt_-sANTXR-Ac2 from a representative experiment. Cargo proteins delivered to the cytosol become biotinylated by cytoplasmic BirA and are subsequently stained with Streptavidin IRDye 680LT. Cytosolic uptake was determined by normalizing signal intensities of cytosolic uptake to the signal of the unspecific interaction of streptavidin with HSP70, which correlates well with the actin signal, and thus can serve as an intrinsic calibration [6]. The delivered cargo DARPin with one internal repeat (labeled “NI_1_C”) is used as a control for maximum signal intensity of cytosolic uptake and cells without any delivered cargo (labeled “Cells”) as a negative control. The location of the mutations in the destabilized (dest) DARPins is listed in Table 1, Figure S3b, and [6].

We first set out to investigate the translocation potential of a set of DARPins. Confirming earlier observations, the consensus DARPins with one and two internal repeats, as well as mutations of the latter, get translocated to the cytosol (Figure 1, Figures S1a–j and S2a–e). An LF_N_ fusion construct designed with two identical NI_1_C DARPins in tandem, LF_N_-NI_1_C-NI_1_C, also showed cytosolic delivery. The consensus DARPin with three internal repeats does not show a signal for cytosolic localization, while destabilizing mutations (NI_3_C dest1–5) restore efficient translocation, as shown previously [6]. The location of the destabilizing mutations is listed for convenience in Table 1 and Figure S3a,b.

Several LoopDARPins and DARPins, all consisting of three internal repeats (N3C format), were tested. DARPins 012_F12, 008_C6, 003_C9, off7, and a destabilized version of it (off7 dest1, carrying the same mutations as NI_3_C dest1; Table 1) were translocated to the cytosol. In contrast, the consensus DARPin NI_3_C was not translocated, nor DARPin 3G124, selected to bind GFP [24], nor was DARPin J1_2_32, specific for c-Jun N-terminal kinases (JNKs) [25]. However, translocation was observed for the JNK binder DARPin J1/2_2_25 with two internal repeats (N2C format).

Figure 1 shows varying cytosolic signal intensities for the different DARPins. Cytosolic degradation, among other factors, even in the presence of the proteasome inhibitor MG-132, might influence the strength of cytosolically detected DARPin signals. Therefore, the BirA assay is used here to distinguish between successful cytosolic translocation and endosomal entrapment of cargo molecules, but not for a direct quantitative comparison of cytosolic cargo concentrations, as discussed further below.

### 3.2. Denaturant-induced Equilibrium Unfolding of DARPin Cargoes

**Figure 2:**
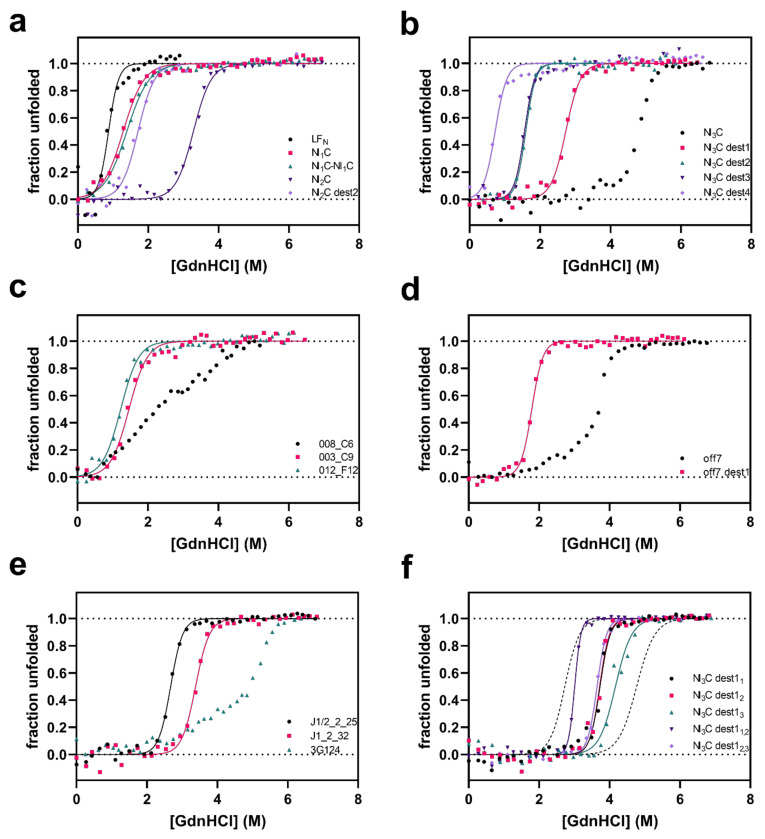
GdnHCl-induced equilibrium unfolding of various cargo DARPins in PBS (pH 7.4) at 20 °C, analyzed by CD spectroscopy. Curves represent a fit to Equation (1). (**a**) LF_N_ and the consensus DARPins NI_1_C, NI_1_C-NI_1_C, NI_2_C, and NI_2_C dest2; (**b**) NI_3_C consensus DARPin and mutated variants of it (NI_3_C dest1–4); (**c**) target-selected LoopDARPins; (**d**) MBP-binding DARPin off7 and a destabilized off7 variant; (**e**) JNK-binding DARPins and eGFP-binding DARPin 3G124; (**f**) NI_3_C dest1 variants with repeat-specific mutations: dashed lines represent NI_3_C with no mutations (right) and NI_3_C dest1 with all mutations (left). Destabilizing mutations (dest1–4) have been described before [6], and their location is listed in Table 1.

Based on the observation that three DARPins in N3C format (NI_3_C, J1_2_32, and 3G124) are not translocated via PA_wt_-sANTXR-Ac2, we investigated the equilibrium thermodynamic stability of the cargo DARPins by denaturant-induced unfolding. For this purpose, the DARPins were incubated at 20 °C in PBS pH 7.4 in increasing concentrations of guanidine hydrochloride (GdnHCl), and their unfolding was monitored using circular dichroism (CD) at 222 nm. Figure 2a–f shows respective unfolding curves for the tested cargo DARPins. Thermodynamic parameters of the cargo DARPins were analyzed assuming two-state unfolding and fitting Equation (1) to the data. The calculated ΔG_0_, m-value, and the denaturation midpoint concentration (*D_m_*) are summarized in Table 1. DARPins 008_C6, off7, NI_3_C, and 3G124 are not well described by two-state equilibria; therefore, we do not report a ΔG_0_ and m-value. For very stable N3C DARPins, this fact has been explained by the C-cap unfolding already at lower denaturant concentrations than the main transition [26], and for a LoopDARPin, it is possible that the cooperativity between the ankyrin repeats is interrupted by the loop insertion. It should be noted that in the present study, the stabilized C-caps [26] were not used, as too high stability of the cargo was not desired.

**Table 1:** Parameters characterizing different cargo DARPins and LF_N_.

Protein	Framework Mutation ^a^	IR ^b^	MW	*D_m_*	ΔG_0_	m	CL ^c^	Ref.
			[kDa]	[M]	[kcal mol^−1^]	[kcal mol^−1^ M^−1^]		
LF_N_	-	-	32.4	0.9	3.6	4.1	Y	[27]
NI_1_C	-	1	11.1	1.3	2.5	2.0	Y	[28]
NI_1_C-NI_1_C	-	1-1	21.0	1.4	2.5	1.8	Y	-
NI_2_C	-	2	14.6	3.3	7.5	2.3	Y	[28]
NI_2_C dest2	(L8A)_1,2,3_	2	14.5	1.7	4.2	2.5	Y	[6]
J1/2_2_25	-	2	14.6	2.7	9.1	3.4	Y	[25]
NI_3_C dest1	(L24A)_1,2,3_	3	18.0	2.7	7.6	2.8	Y	[6]
NI_3_C dest1_1_	(L24A)_1_	3	18.1	3.7 *	-	-	N	-
NI_3_C dest1_2_	(L24A)_2_	3	18.1	3.7 *	-	-	N	-
NI_3_C dest1_3_	(L24A)_3_	3	18.1	4.2 *	-	-	N	-
NI_3_C dest1_1,2_	(L24A)_1,2_	3	18.0	3.0 *	-	-	Y/N	-
NI_3_C dest1_2,3_	(L24A)_2,3_	3	18.0	3.6 *	-	-	N	-
NI_3_C dest2	(L24G)_1,2,3_	3	17.9	1.6	6.7	4.2	Y	[6]
NI_3_C dest3	(L8A, L24A)_1,2,3_	3	17.8	1.6	6.4	4.1	Y	[6]
NI_3_C dest4	(L8G, L24A)_1,2,3_	3	17.8	0.7	2.6	3.5	Y	[6]
008_C6	-	3	18.2	2.1 *	-	-	Y	[29]
003_C9	-	3	18.3	1.5	3.3	2.2	Y	[29]
012_F12	-	3	18.1	1.2	2.8	2.3	Y	[29]
off7	-	3	18.1	3.6 *	-	-	Y	[21]
off7 dest1	(L24A)_1,2,3_	3	18.0	1.8	6.7	3.7	Y	-
NI_3_C	-	3	18.1	4.8 *	-	-	N	[28]
J1_2_32	-	3	18.0	3.4	10.1	3.0	N	[25]
3G124	-	3	17.9	5.2*	-	-	N	[24]

ΔG_0_ and m were calculated from fits of Equation (1) to GdnHCl-induced equilibrium unfolding curves. *D_m_* was calculated using Equation (2). A two-state fit model could not reasonably be fitted to the DARPin data marked with an asterisk (*); therefore, *D_m_* was estimated based on the main unfolding step. Cytosolic localization was determined with the BirA assay. ^a^ Repeat-specific numbering; ^b^ Number of internal repeats (IR); ^c^ Cytosolic localization (CL) measured via BirA assay.

LF_N_ (not fused to another protein) is translocated to the cytosol. The individual LF_N_ subunit has a *D_m_* of 0.9 M GdnHCl and a ΔG_0_ value of 3.6 kcal mol^−1^. The smallest DARPin, NI_1_C, shows the highest signal for cytosolic localization and has the lowest ΔG_0_ value, 2.5 kcal mol^−1^. The NI_1_C-NI_1_C fusion, the DARPin cargo tested with the highest nominal MW, has the same protein stability as the single NI_1_C DARPin, showing that the two DARPins are indeed unfolding independently as expected, and the fusion protein is efficiently translocated (Figure 1 and Figure 2a, Table 1).

As described previously [28], the thermodynamic stability of DARPins increases with the increasing number of internal repeats (Table 1). For DARPins with two internal repeats (NI_2_C, NI_2_C dest1–6, and the selected binder J1/2_2_25), cytosolic localization can be detected for all the tested constructs (Figure 1). Even the stable NI_2_C and J1/2_2_25, with ΔG_0_ values of 7.5 kcal mol^−1^ and 9.1 kcal mol^−1^, respectively, can be unfolded and translocated by PA_wt_-sANTXR-Ac2, although potentially with lower efficiency than NI_1_C. Consensus DARPins carrying mutations in the framework (NI_2_C dest1–6) modestly increased cytosolic localization signals. We therefore measured the unfolding curves for only one of the destabilized NI_2_C DARPins, verifying that the framework mutations would further reduce the stability, as described below for mutations introduced in the NI_3_C consensus DARPin (Figure 2a,b), but because the stable NI_2_C is translocated, ΔG_0_ values of further NI_2_C mutants would bring no further information on translocation efficiency.

The NI_3_C consensus DARPin is not translocated to the cytosol (Figure 1, Figure S1c,d) [6] and shows a *D_m_* of 4.8 M GdnHCl, but because it does not follow two-state unfolding, its ΔG_0_ cannot be determined from attempting such a fit [26]. The destabilizing mutations introduced in the NI_3_C consensus DARPin (NI_3_C dest1–6, [6]) significantly lowered *D_m_* and ΔG_0_, and for these mutants, two-state unfolding seems to describe the unfolding (Figure 2b, Table 1).

The introduction of single mutations in each of the three internal repeats (either L24A in NI_3_C dest1 or L24G in NI_3_C dest2) reduced the stability of the DARPin to ΔG_0_ values of 7.6 kcal mol^−1^ and 6.7 kcal mol^−1^, respectively. When two mutations are introduced in each of the three internal repeats (combining L8A and L24A in NI_3_C dest3 or L8G and L24A in NI_3_C dest4), a further decreased DARPin stability was measured, with a ΔG_0_ of 6.4 kcal mol^−1^ for the NI_3_C dest3 or a ΔG_0_ of 2.6 kcal mol^−1^ for the NI_3_C dest4. The latter is already equivalent to the level of a DARPin with a single internal repeat (Table 1).

The target-selected binders were obtained from libraries of N2C, N3C, and N3C LoopDARPin formats and thus differ in the target-binding surface and stability (Figure 2c,d). DARPins J1_2_32 and 3G124 were not translocated to the cytosol and showed an equilibrium stability comparable to the consensus NI_3_C DARPin (Table 1). Having undergone target selection, therefore, does not necessarily decrease ΔG_0_ to the point to allow translocation through the pore formed by PA_wt_-sANTXR-Ac2. Nonetheless, many N3C binders were translocated (008_C6, 003_C9, 012_F12, off7) without further modification, and if such a further destabilization was carried out (off7 dest1), it did not further affect translocation. The JNK-binding N2C DARPin J1/2_2_25 was translocated without modification, which may not be surprising, as the stable consensus NI_2_C was translocated as well.

Because the introduction of a mutation in all three repeats of the NI_3_C consensus DARPin backbone restored delivery to the cytosol, it was of interest to investigate whether mutations in only one or two of the repeats might already be sufficient. We therefore created variants of the NI_3_C dest1 with the L24A mutation only in the first (denoted with a subscript, NI_3_C dest1_1_), second (NI_3_C dest1_2_), or third (NI_3_C dest1_3_) repeat, or with two L24A mutation in the first and second (NI_3_C dest1_1,2_) or in the second and third (NI_3_C dest1_2,3_) internal repeat. We found that efficient cytosolic delivery was not restored with these variants, and only the variant NI_3_C dest1, with all three internal repeats carrying the L24A mutation, was detected in the cytosol (Figure 1, Figure S4a–c). We determined the equilibrium stability of these mutated variants via denaturant-induced unfolding measured by CD. As expected, we found them to be in between the variant NI_3_C dest1, which is destabilized in all three internal repeats, and the consensus NI_3_C (Figure 2f, Table 1). These findings indicate that all three internal repeats need to be mutated to restore efficient delivery for the very stable NI_3_C consensus DARPin, but the intermediate unfolding curves also show that a more subtle destabilization is possible, and a particular binder may be rescued by such a small change.

One attractive feature of the DARPin structure is that these destabilizing mutations can be introduced in the framework, away from target-contacting residues. Nonetheless, we tested whether mutating all three internal repeats in these non-contacting residues would influence target binding by measuring the binding kinetics by SPR. We compared the DARPin off7 and the destabilized variant off7 dest1, carrying the same mutations as the DARPin NI_3_C dest1 (Table 1). We determined a similar K_D_ value for off7 dest1 (K_D_ = 2.45 nM) compared to off7 (K_D_ = 3.07 nM) without any mutation (Figure S5a,b, [21]), confirming that mutations in the backbone of the DARPin do not influence its binding affinity. Therefore, the DARPin structure permits one to introduce destabilizing mutations (Figure 2d, Table 1) that still maintain the structure of the target-binding interface, as they do not decrease target-binding affinity, and thus stability-determining residues and affinity-determining residues can indeed be separated from each other.

Anthrax toxin translocates its cargo molecules through the endolysosomal membrane to the cytosol. One of the driving forces of the transport mechanism is the pH difference between the late endosomal compartment, around pH 6.0, and the neutral pH in the cytosol. We therefore tested the DARPin integrity at different pH values, with a subset of DARPins (NI_1_C, NI_2_C, NI_3_C, J1/2_2_25, and J1_2_32) and observed no changes in alpha-helical content, for any DARPin tested, from pH 7.4 to pH 6.5 or pH 6.0 (Figure S6a–c). By further decreasing the pH to pH 5.5 and finally stepwise down to pH 3.5, we observed partial precipitation of these proteins that all have a low isoelectric point, and eventually, all DARPins also showed a reduced alpha-helical structure (Figure S6d–h). This is probably due to the critical histidine in the TLPH motif, which is part of every consensus ankyrin repeat and highly conserved in ankyrins [22].

In order to further evaluate the influence of pH on the equilibrium unfolding behavior of the cargo DARPins, the previously mentioned set of DARPins, additionally including NI_3_C dest1, were tested for their equilibrium unfolding at pH 6.0 (Figure S7a). Their *D_m_* was calculated, and all tested constructs showed very similar unfolding curves at pH 6.0 and pH 7.4 (Table 1, Figure S7b).

We further tested the equilibrium unfolding behavior of LF_N_-DARPin fusion constructs (Figure S8a) to confirm the independent unfolding of the LF_N_ domain, and the cargo DARPin. LF_N_-NI_2_C and LF_N_-NI_3_C fusion constructs confirm the independent unfolding of the fusion partners. LF_N_ unfolds in both fusion constructs at a *D_m_* of 0.9 M (Figure S8b), similar to the non-fused LF_N_ (Table 1). For the consensus DARPins with two and three internal repeats, a *D_m_* of 3.5 M and 4.9 M (Figure S8b) was observed, similar to the *D_m_* of the DARPins alone (Table 1). For the LF_N_-NI_1_C fusion protein, a two-state unfolding behavior was observed. Even though it is expected that LF_N_ and NI_1_C DARPin will also unfold independently, they share a similar *D_m_* (1.0 M), and thus the curves will be indistinguishable (Table 1, Supplementary Figure S8).

Having characterized various cargo DARPins for uptake and stability, we tested the correlation between the normalized cytosolic signal intensity, shown in Figure 1, as well as the *D_m_* and ΔG_0_ (Table 1). Figure S9a,b confirms the overall trend that the more stable a cargo DARPin is, the less efficiently it will be delivered. However, there are several factors that contribute to deviations from this correlation. Because of the large number of samples, we have to cross-compare Western blots, with increased uncertainties of quantification. Furthermore, we are observing steady-state levels, and thus varying cytosolic degradation rates will also impact the observed cytosolic levels.

## 4. Discussion

In this study, we provide an in-depth quantitative investigation of the relationship between the potential to be delivered to the cytosol through the pore formed by PA_wt_-sANTXR-Ac2 and the equilibrium thermodynamic stability of various DARPins. For both properties, we tested consensus DARPins that had shown translocation in a previous study [6] and further expanded the range of cargoes to now include target-selected DARPins, target-selected LoopDARPins, and a two-DARPin fusion cargo construct.

We confirmed our previous findings, showing that all DARPins with one or two internal repeats are translocated to the cytosol of Flp-In 293-EpCAM-BirA cells overexpressing EpCAM, using an EpCAM-retargeted PA fusion, PA_wt_-sANTXR-Ac2 [6]. The NI_3_C consensus DARPin with three internal repeats is translocated only upon implementing destabilizing mutations within the DARPin framework. The target-selected DARPins confirmed these findings, as two of the tested N3C DARPins (with three internal repeats) remain trapped in the endosome, while one did translocate, as did all target-selected LoopDARPins with three internal repeats, as well as the above-mentioned DARPin with two internal repeats.

In an earlier study, we hypothesized that lower thermodynamic stability of the DARPin leads to a higher translocation up to a cutoff stability point. Destabilization lower than this cutoff would not lead to higher translocation efficiency [6] (see below). However, this threshold stability had not been measured. Therefore, we tested whether the DARPin equilibrium stability could predict the ability of the anthrax pore to translocate cargo molecules. Because LF_N_ has naturally evolved to get translocated and itself has a low equilibrium stability, and because the cargo unfolds independently in a fusion with LF_N_, as found by the identical equilibrium denaturation curves, the cargo DARPin alone determines the translocation. From the denaturant-induced equilibrium unfolding curves, we could correlate stability and cytosolic translocation and found that only molecules having a ΔG_0_ value of less than 10 kcal mol^−1^ showed a cytosolic signal. In contrast, molecules with higher stability than this value, such as three of the N3C DARPins tested, showed no translocation. This ΔG_0_ value is not reached by N1C or N2C molecules, as with the increasing number of repeats, the stability of the DARPin increases [28], and thus all N1C or N2C molecules were translocated.

A previous study further tested the unfolding rates of DARPin molecules, which decreased with the increasing number of internal repeats [28]. This unfolding rate of cargo molecules might be an additional factor influencing cargo translocation, as endosomal degradation might reduce the time available to unfold and translocate within the endo-/lysosomal compartment. However, we currently do not have DARPins with the same equilibrium ΔG_0_ but different folding and unfolding rates, and we cannot yet distinguish kinetic and equilibrium effects. Because equilibrium unfolding of the tested DARPins at an endosomal-like pH did not change the *D_m_* of the DARPins, we deduce that our measurements at neutral pH are relevant to describe the relative stability of the DARPins at endosomal pH.

We hypothesized earlier [6] that DARPin library members with randomized residues can be less stable than consensus DARPins and could thus get translocated without destabilizing mutations even in the N3C format, as the consensus DARPins were specifically designed and optimized for their stability [6,28,30]. Nonetheless, target-selected binders were not necessarily less stable than their ’parental’ consensus variant [30]. Selected binders for JNKs were available in both N2C and N3C format, and the N2C variants could be delivered, while the N3C variant was too stable to get translocated [25,31]. Rational destabilization of target-selected binders might thus be necessary for efficient translocation for some N3C variants, and we showed that this can be achieved without losing target affinity. We showed that eGFP was not translocated, and we thus used it as a negative control. It forms a β-barrel structure, and previous studies have reported ΔG_0_ values for eGFP above 10 kcal mol^−1^ [32], which is in the range of those DARPin cargoes that were not translocated.

We could confirm that the size of the cargo DARPins does not limit translocation, at least in the ranges tested. Fusing two NI_1_C DARPins to each other and to LF_N_ resulted in cytosolic translocation, even though this NI_1_C-NI_1_C fusion molecule is 3 kDa (MW_calc_) larger than the NI_3_C consensus DARPin, but it consists of independently unfolding domains. These findings complement the results from our previous study, where we started destabilizing the NI_2_C and NI_3_C consensus DARPin to show that stability might be more important than size [6]. The equilibrium unfolding behavior of LF and LF_N_ has been tested before with differing results, depending on the buffer, pH, and fits used [33,34,35]. For LF_N_, we measured a ΔG_0_ value of 3.6 kcal mol^−1^. We could confirm an uptake of proteins with much higher stability than LF_N_, up to 10 kcal mol^−1^; however, the maximum size of a cargo protein remains currently unknown. We propose that any protein below the stability threshold, devoid of disulfide bridges and not larger than LF itself (90 kDa), is likely to get translocated.

Another important factor to consider in destabilizing cargo molecules is the subsequent cytosolic refolding and stability of the protein in the cytosol. NI_3_C dest4 has two mutations per internal repeat and is destabilized by more than would be needed for translocation. If these mutations are introduced into a DARPin that recognizes a cytosolic target, it might become too unstable to refold and to have a biologic effect in the cytosol and instead be more prone to a faster proteolytic degradation in the cytosol. A suitable assay for cytosolic refolding is therefore in high demand and currently under development. We thus propose that for optimal intracellular binding activity, there is a certain window of opportunity, characterized by maximum stability that allows unfolding in the endosome concomitant with transport and minimum stability needed for efficient refolding in the cytosol to escape proteolytic degradation.

## 5. Conclusions

The stability translocation threshold of anthrax-toxin-mediated delivery is correlated to the equilibrium protein stability of the cargo, measured via denaturant-induced unfolding. Combining our results from the biotin ligase assay and these thermodynamic stability measurements, we identified a threshold range at about 10 kcal mol^−1^, above which cytosolic translocation of DARPin cargoes becomes essentially undetectable. The measurement of the DARPin stability therefore enables the design of translocatable DARPins for cytosolic targets in a high-throughput manner, without having to test each individual one for its delivery in an initial screening. Furthermore, mutations can be introduced to destabilize the DARPin just enough for translocation, while target binding can be maintained.

## Data Availability

All data are included in this manuscript and its supplementary files.

## Supplementary Materials

The following are available online at https://www.mdpi.com/article/10.3390/pharmaceutics13081285/s1, Figure S1: Western blot and quantification showing delivery of different LF_N_-cargo constructs with PA_wt_-sANTXR-Ac2, Figure S2: Total cellular uptake measured via HA-tag of the LF_N_-cargo, Figure S3: Destabilizing mutations in DARPin framework, Figure S3: Western blot and quantification showing delivery of different LF_N_-cargo constructs of the consensus DARPin NI_3_C and variants of the NI_3_C dest1 with PAwt-sANTXR-Ac2, Figure S4: Western blot and quantification showing delivery of different LF_N_-cargo constructs of the consensus DARPin NI_3_C and variants of the NI_3_C dest1, Figure S5: SPR measurement of off7 and off7 dest1, Figure S6: pH titration of different DARPins, Figure S7: GdnHCl-induced equilibrium unfolding at pH 6 of NI_1_C, NI_2_C, NI_3_C, NI_3_C dest1, J1/2_2_25 and J1_2_32, Figure S8: GdnHCl-induced equilibrium unfolding of LF_N_-NI_1_C, LF_N_-NI_2_C and LF_N_-NI_3_C in PBS (pH 7.4) at 20 °C analyzed by CD spectroscopy, Figure S9: Correlation of normalized cytosolic update intensity of LF_N_-DARPin cargo and the denaturation midpoint or ΔG_0_ of the DARPin.

## Abbreviations

PA	protective antigen
DARPin(s)	designed ankyrin repeat protein(s)
CD	circular dichroism
LF	lethal factor
EF	edema factor
AEBSF	4-(2-aminoethyl) benzenesulfonyl fluoride
IMAC	immobilized metal ion affinity chromatography
CV	column volume
MRE	mean residue ellipticity
*D_m_*	denaturation midpoint
SPR	surface plasmon resonance
EpCAM	epithelial cell adhesion molecule
LF_N_	lethal factor 1–254
BirA	biotin ligase derived from *E. coli*
dest	destabilized
JNKs	c-Jun N-terminal kinases
GdnHCl	guanidine hydrochloride
IR	internal repeats
CL	cytosolic localization
