# Supplementary material for: Thermodynamic Stability Is a Strong Predictor for the Delivery of DARPins to the Cytosol via Anthrax Toxin
Source: Pharmaceutics. 2021 Aug 18;13(8):1285. doi: 10.3390/pharmaceutics13081285 (PMC8401532; doi:10.3390/pharmaceutics13081285)
Supplement: Supplementary file 1 [file pharmaceutics-13-01285-s001.zip › pharmaceutics-1289895-supplementary.pdf]

pharmaceutics

Supplementary information

Thermodynamic Stability Is a Strong Predictor for Delivery of DARPins to the Cytosol via Anthrax Toxin

Lukas Becker [1], Jasleen Singh Badwal [1], Fabian Brandl [1], Wouter P. R. Verdurmen [2] and Andreas Plückthun [1,*]

[1]Dept. of Biochemistry, University of Zurich, Winterthurerstr. 190, 8057 Zurich, Switzerland
[2]Dept. of Biochemistry, Radboud Institute for Molecular Life Sciences (RIMLS), Radboud University Medical Center, Geert Grooteplein 28, 6525 GA, Nijmegen, The Netherlands
* Correspondence: correspondence to plueckthun@bioc.uzh.ch

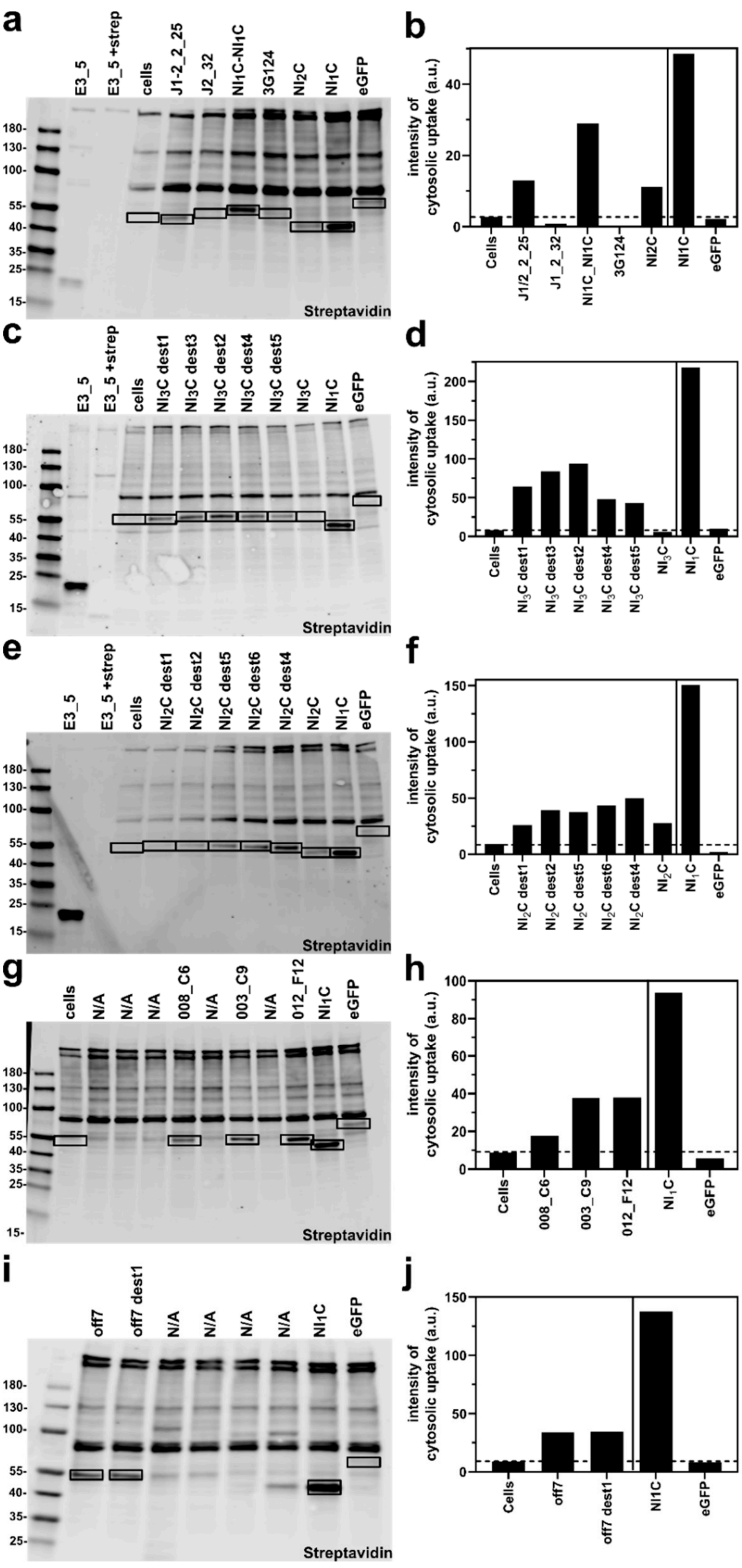

Figure S1. Western blot and quantification showing delivery of different LF_N-cargo constructs with PA_{wt}-sANTXR-Ac2. (a, c, e, g, i) Cargo proteins delivered to the cytosol are biotinylated by cytoplasmic BirA and stained with Streptavidin IRDye 680LT (b, d, f, h, j) Quantification of Western blot bands from (a, c, e, g, i). The dashed lines represent background signals (i.e. cells only). NI_1C and eGFP are used as delivery controls for maximum signal intensity of cytosolic uptake (NI_1C) and no cytosolic localization (eGFP). Destabilized (dest) DARPins have been described previously [1] and the location of mutations is listed in Table 1 in the main text and Supplementary Figure S3.

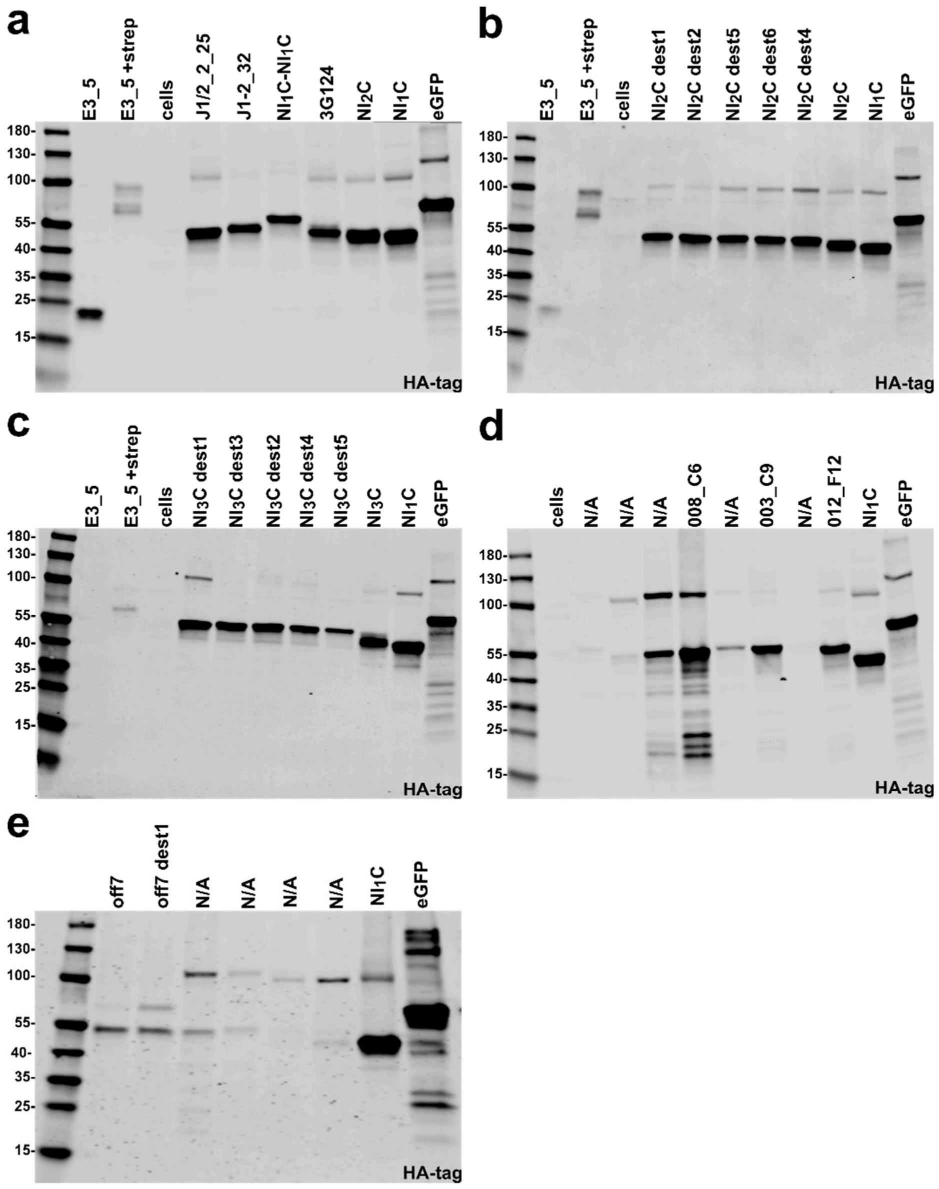

Figure S2. (a-e) Total cellular uptake measured via HA-tag of the LF$_N$-cargo. This corresponds to the samples of the BirA assay in Figure S1. Destabilized (dest) DARPins (b, c) have been described previously [1] and the location of mutations is listed in Table 1 in the main text and Supplementary Figure S3.

a

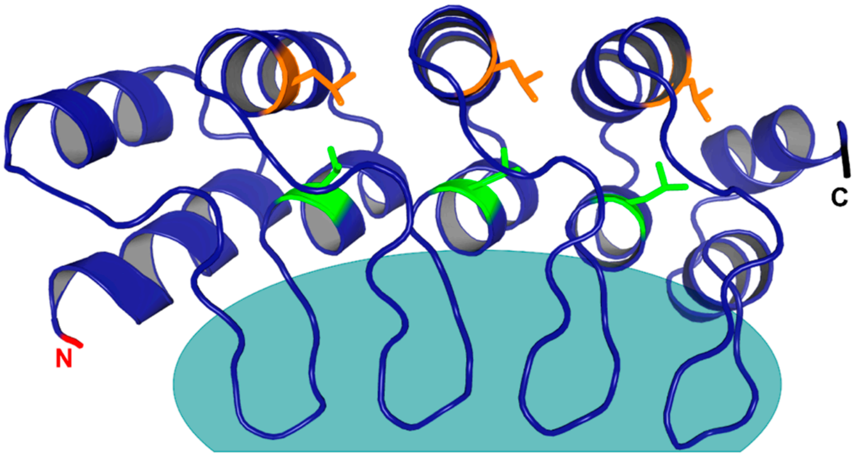

b

DARPin	Mutation(s)	DARPin	Mutation(s)
NI_2C dest1	L8G	NI_3C dest1	L24A
NI_2C dest2	L8A	NI_3C dest2	L24G
NI_2C dest4	L23A	NI_3C dest3	L8A, L24A
NI_2C dest5	L24G	NI_3C dest4	L8G, L24A
NI_2C dest6	L24A	NI_3C dest5	L8A, L24G

Figure S3. Destabilizing mutations in DARPin framework as published previously [1]. (a) Positions of framework mutation of each internal repeats are represented in green (L8) and orange (L24). The respective DARPin target binding area is represented by the grey area, spatially distant to the framework mutations. N and C termini are labelled in red or black, respectively; structural representation adapted from PDB ID 1svx. (b) Position and amino acid substitution of destabilized DARPins of each internal repeat; each position was mutated in all three internal repeats.

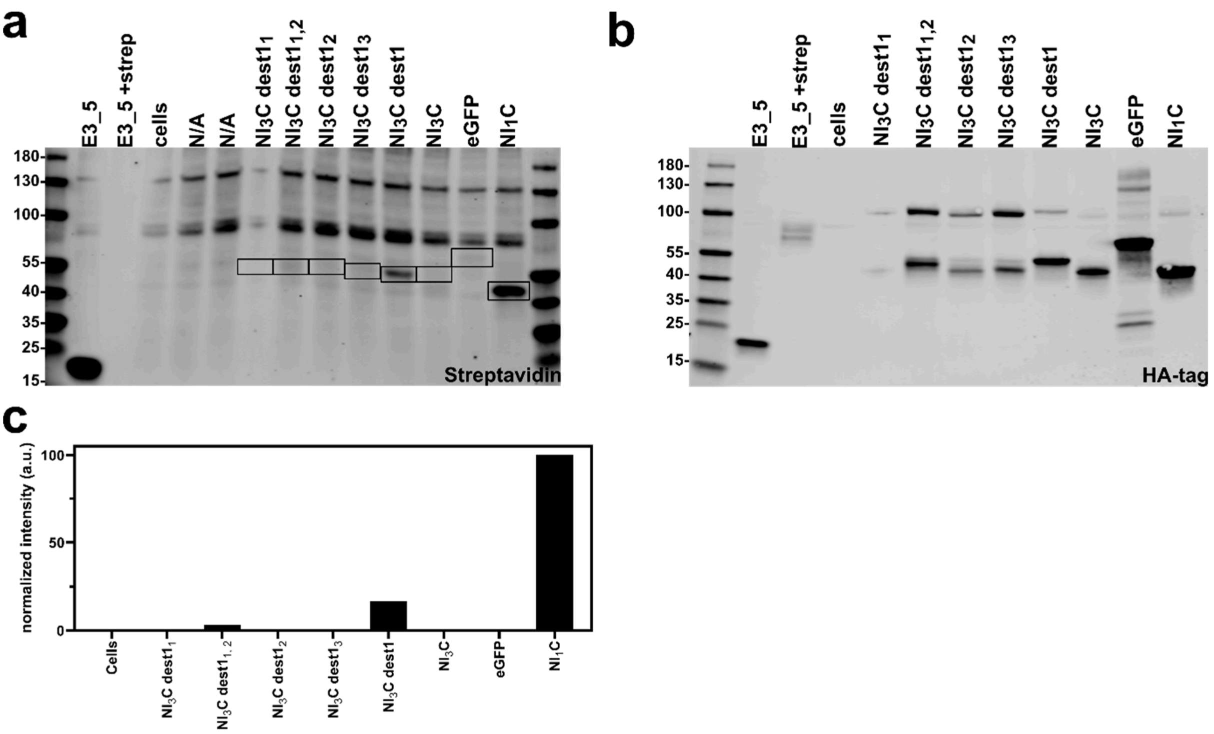

Figure S4. Western blot and quantification showing delivery of different LF_N-cargo constructs of the consensus DARPin NI_3C and variants of the NI_3C dest1 with PA_{wt}-sANTXR-Ac2. Variants with the L24A mutation are mutated only in the first (NI_3C $dest1_1$), only the second (NI_3C $dest1_2$), only the third (NI_3C $dest1_3$), first and second (NI_3C $dest1_{1,2}$) or second and third (NI_3C $dest1_{2,3}$) internal repeat. Western Blots (a, b) and respective quantification (c) of the biotin ligase assay, showing efficient cytosolic translocation only for the fully destabilized variant NI_3C dest1.

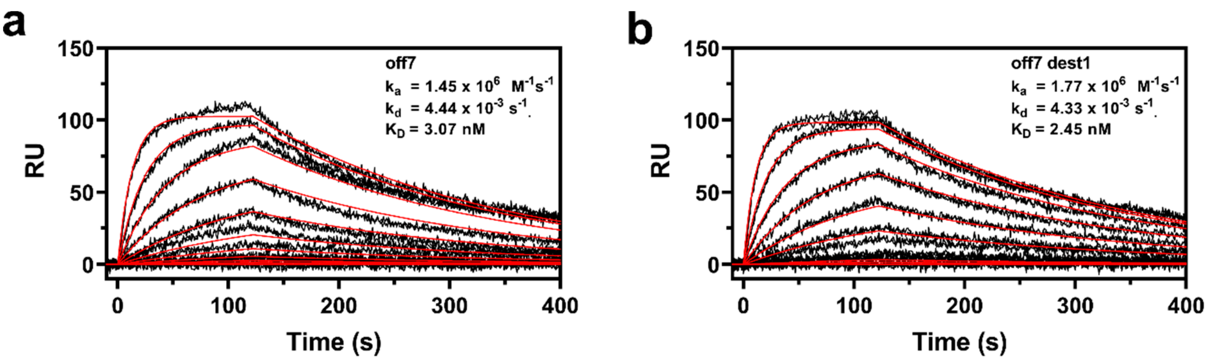

Figure S5. SPR measurement of off7 and off7 dest1 (L24A mutation in all three internal repeats) showing a similar K_D for target binding.

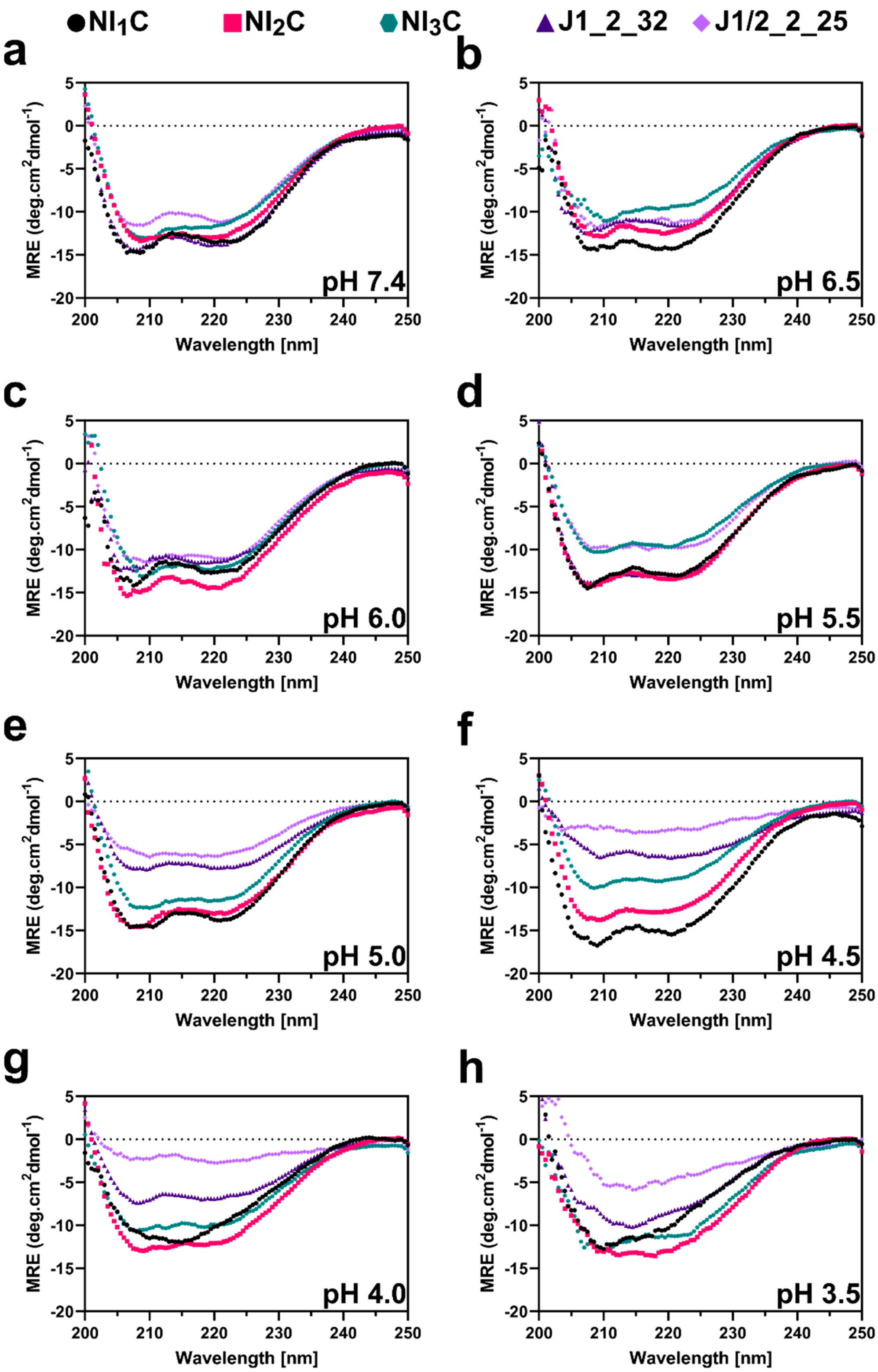

Figure S6. pH titration of different DARPins. NI$_1$C, NI$_2$C, NI$_3$C, J1/2_2_25 and J1_2_32 were incubated overnight in PBS at pH 7.4 (a), MES at pH 6.5 (b), MES at pH 6.0 (c), sodium acetate at pH 5.5 (d), sodium acetate at pH 5.0 (e), sodium acetate at pH 4.5 (f), sodium acetate at pH 4.0 (g), citric acid at pH 3.5 (h); all buffers were used at 50 mM and supplemented with 150 mM NaCl, except PBS. Subsequently, proteins were analyzed via circular dichroism (CD).

a

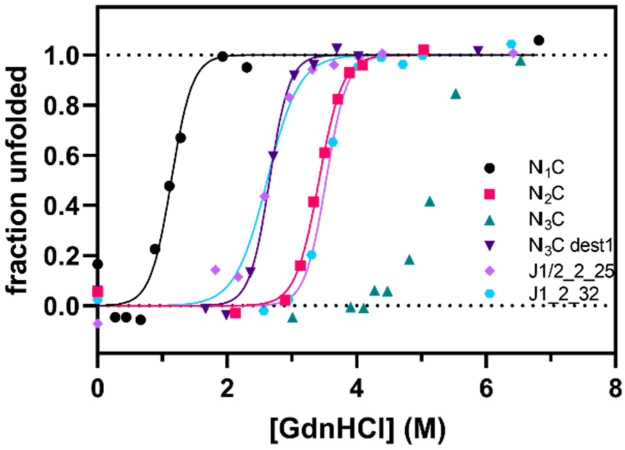

b

Protein	D_m [M] pH 7.4	pH 6.0
NI$_1$C	1.3	1.1
NI$_2$C	3.3	3.4
NI$_3$C	4.8*	5.2*
NI$_3$C dest1	2.7	2.7
J1/2_2_25	2.7	2.6
J1_2_32	3.4	3.5

Figure S7. GdnHCl-induced equilibrium unfolding at pH 6 of NI$_1$C, NI$_2$C, NI$_3$C, NI$_3$C dest1, J1/2_2_25 and J1_2_32 in 50 mM MES (pH 6.0) supplemented with 150 mM NaCl at 20 °C analyzed by CD spectroscopy; (b) Denaturation midpoints (D_m) were taken from Table 1 (PBS, pH 7.4) and by fitting Equation 1 to the data from (a). Values marked with an asterisk (*) were estimated by a nonlinear fit (GraphPad Prism 8.0, X is concentration) to experimental data since fitting Equation 1 was not possible due to too few datapoints.

a

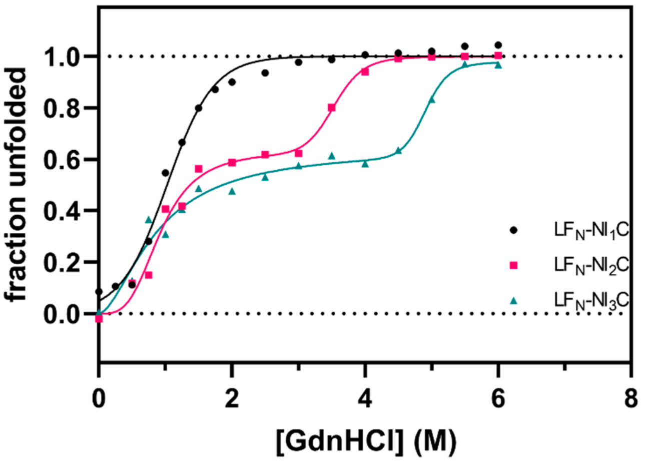

b

Protein	$D_{m,1}$ [M]	$D_{m,2}$ [M]
LF_N-NI_1C	1.0	-
LF_N-NI_2C	0.9*	3.5*
LF_N-NI_3C	0.9*	4.9*

Figure S8. (a) GdnHCl-induced equilibrium unfolding of LF_N-NI_1C, LF_N-NI_2C and LF_N-NI_3C in PBS (pH 7.4) at 20 °C analyzed by CD spectroscopy. (b) The denaturation midpoint ($D_{m,1}$) for LF_N-NI_1C was determined by fitting Equation 1 to the data obtained in (a). The two denaturation midpoints $D_{m,1}$ and $D_{m,2}$ of LF_N-NI_2C and LF_N-NI_3C, marked with an asterisk (*), were estimated by fitting a nonlinear biphasic fit (GraphPad Prism 8.0) to the data.

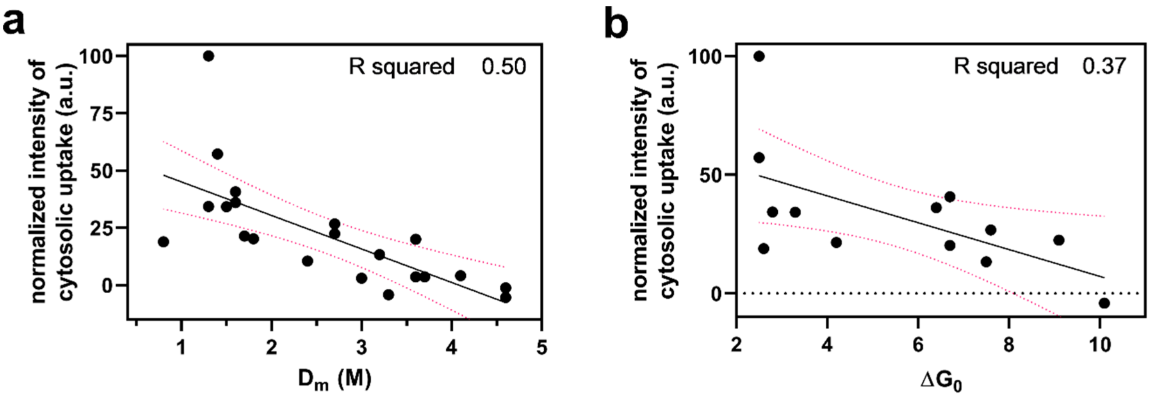

Figure S9. Correlation of normalized cytosolic update intensity of LFN-DARPin cargo and the denaturation midpoint (a) or ΔG_0 (b) of the DARPin. The coefficient of determination R^2 was calculated using GraphPad Prism 8.0.
